# Anterior Controllable Antedisplacement and Fusion (ACAF) for Cervical Stenosis Patients With Hyperextension Injury: A Retrospective Study

**DOI:** 10.1111/os.14319

**Published:** 2024-12-12

**Authors:** Shuangxi Sun, Yingying Miao, Tao Xu, Kaiqiang Sun, Yijuan Lu, Jingchuan Sun, Jiuyi Sun, Jiangang Shi

**Affiliations:** ^1^ Department of Orthopedic Surgery The Center Hospital of Weihai City Weihai Shandong China; ^2^ Department of Orthopedic Surgery Changzheng Hospital, Navy Medical University Shanghai China; ^3^ Department of Orthopedic Surgery Hospital of the People's Liberation Army Ningbo Zhejiang China; ^4^ Department of Orthopedics Naval Medical Center of PLA, Navy Medical University Shanghai China; ^5^ Department of Radiology The Second Affiliated Hospital of Naval Medical University Shanghai China

**Keywords:** acute central cord syndrome, anterior controllable antedisplacement and fusion, clinical outcomes, hyperextension injury

## Abstract

**Objective:**

Central cord syndrome (CCS) is an incomplete spinal cord injury (SCI) causing severe motor weakness, and timely decompression via surgical intervention facilitates better recovery. Anterior controllable antedisplacement and fusion (ACAF) is a novel decompression technique and achieved satisfactory outcomes in treating cervical degenerated diseases. However, the clinical effects of ACAF on CCS remains unknown. This present study aimed to investigate the clinical outcomes of ACAF for cervical stenosis patients with CCS due to hyperextension injury.

**Methods:**

This is a retrospective study, and patients who underwent ACAF due to CCS in our institution from July 2021 to December 2022 were enrolled based on the inclusion and exclusion criteria. All patients underwent x‐ray, computed tomography (CT), and magnetic resonance imaging (MRI) before and after surgery. The duration of follow‐up was at least 12 months. The radiological parameters included associated pathologies, prevertebral hyperintensity (HI), intramedullary signal intensity (ISI), and Torg–Pavlov ratio (TPR). The cervical stability was also evaluated. Neurological function was assessed using the American Spinal Injury Association (ASIA) grading system and Japanese Orthopaedic Association (JOA) score. The Mann–Whitney *U* test was used to compare the clinical outcomes preoperatively and postoperatively.

**Results:**

Finally, 13 patients (7 male and 6 female) with the minimum of 12‐month follow‐up were finally enrolled in this study, with the mean age of 56.6 ± 12.5 years (range, 39–74 years). There were eight patients suffered CCS due to fall, three due to vehicle accident, and two due to diving injuries. The average delay from injury to surgery was 2.23 days (range, 1–4 days), and the mean duration of follow‐up was 16.1 ± 3.5 months. In terms of prevertebral HI and ISI, C4–C6 were the most affected region. In addition, 76.9% (10 of 13) patients were observed to have cervical stenosis indicated by TPR. Associated pathologies were herniated nucleus pulposus (HNP) in five patients, OPLL in three cases, and HNP‐osteophyte complexes (HNP‐OC) in six patients. At the final follow‐up, 13 patients were improved to E. The mean JOA score improved to 15.4 ± 1.0, with the recovery rate of 77.0% ± 12.0%. Two patients experienced postoperative dysphagia, two patients had hoarseness, and one patient suffered postoperative hematoma.

**Conclusions:**

ACAF can be a good option for treating CCS patients due to hyperextension injury with underlying cervical spondylosis and stenosis.

## Graphical abstract

The key procedures of anterior controllable antedisplacement and fusion (ACAF) in treating cervical stenosis patients with hyperextension injury are as follows: removal of and osteophytes of the affected segments (A), removal of the anterior portion of the involving vertebral bodies and the herniated disc tissues and (B), prefixation of the cervical plate and screws, and slotting on both sides of the affected vertebral bodies (C), controllable antedisplacement of the vertebral body (D). From a perspective of axial view, the procedures can be shown as follows: ossification of posterior longitudinal ligament (OPLL) or osteophytes at the operative level (E), removal of the anterior portion of the vertebral body (F), slotting at one side lateral to the margin of OPLL or osteophytes (G), prefixation of cervical plate and screws (H), slotting at the other side lateral to the margin of OPLL or osteophytes (I), controllable antedisplacement of the vertebral bodies (J).

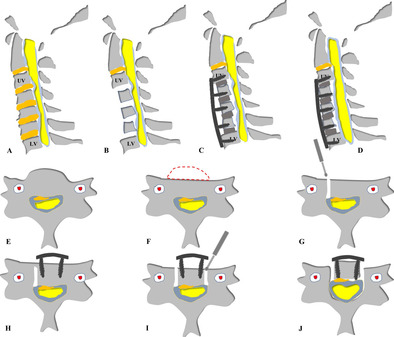

## 1 Introduction

Central cord syndrome (CCS) has ranked as one of the most common types of spinal cord injury (SCI), accounting for 70% of all incomplete spinal cord, and most often occurs in older populations with underlying cervical spondylosis and stenosis triggered by a hyperextension mechanism [1]. It can also occur in young individuals who sustain acute trauma to the cervical spine and, less commonly, as a result of nontraumatic causes. The primary injury, usually resulting from rapid spinal cord compression and contusion, will initiate a signaling cascade of down‐stream events collectively known as secondary injury [2]. This syndrome is characterized by a disproportionately greater loss of motor power in the upper extremities than the lower extremities with varying degrees of sensory loss below the level of the lesion and bladder dysfunction. Given the catastrophic impact of CSS on the individual and society, it has come to a consensus that effective therapies aimed at reducing the extent of tissue destruction and improving neurologic function after the initial CSS are urgently needed.

Conservative treatment was initially recommended, and physical/occupational and corticosteroid therapies have been commonly encouraged [2]. However, the majority of CCS patients did not acquire complete recovery [3]. Surgical management has been suggested in recently years, especially for patients with previous cervical spondylosis or stenosis. Anterior cervical discectomy and fusion (ACDF) can deal with the lesion at disc level with less complications. However, ACDF cannot deal with the compression behind vertebra bodies, such as ossified ligament [4]. Anterior cervical corpectomy and fusion (ACCF) is an effective option for patients with multilevel compression behind the vertebra bodies. However, this technique has relatively higher incidence of complications, such as cerebrospinal fluid (CSF) leakage, hemorrhage, and intraoperative SCI, especially for multilevel corpectomy [5]. Posterior approach can also achieve decompression of neural elements by posterior floating of the cord and maintain cervical movement after surgery. However, movement preservation might not be necessary in patients with cervical instability due to the high‐energy trauma. In addition, for those who have cervical kyphosis or deformation, the surgical effect of simple decompression such as motion‐preserving laminoplasty would be limited, instrumented fusion would be also required [6]. The combined anteroposterior approach is also another clinical choice. Nevertheless, the occurrence of longer surgical duration, larger surgical damage, and postoperative posterior neck pain still jeopardize patients' quality of life and preclude its generalization [7].

Anterior controllable antedisplacement and fusion (ACAF) has been initially proposed for patients with cervical myelopathy due to ossification of the posterior longitudinal ligament (OPLL) and acquired good results [8, 9, 10, 11]. This technique can achieve larger direct decompression including compression behind the vertebral bodies compared with ACDF, and acquire better reconstruction of spinal canal compared with ACCF [10]. In addition, patients treated by ACAF can acquired good restoration of cervical lordosis and spinal cord alignment [11]. In this technique, the affected vertebrae and compressive components are not removed but isolated and moved anteriorly to remodel the anterior column of the cervical spine. Since osteotomized vertebral body used as an autograft with intervertebral cages and cervical plate, cervical spine can acquire satisfactory stability after surgery. However, in spite of satisfactory outcomes of ACAF in treating patients with cervical OPLL, the surgical outcomes of ACAF for patients with CCS due to hyperextension injury remains to be further elucidated.

Therefore, this present study aimed (1) to evaluate the clinical characteristics of cervical stenosis patients with CSS due to hyperextension; (2) to evaluate the radiological characteristics of patients with CSS due to hyperextension; (3) to investigate the clinical efficacy and safety of ACAF for treating patients with CCS due to hyperextension. The results of this study will expand the clinical indication of ACAF, as well as provide a novel alternative for future treatment of cervical stenosis patients with CSS due to hyperextension.

## 2 Methods

The STROBE Reporting Guideline was included in this case series study.

### 2.1 Patients Populations

This is a retrospective study. The medical data of totally 21 patients who underwent ACAF due to CCS in our institution were reviewed, and the recruitment period was from July 2021 to December 2022 based on the admission time of CCS patients.

The inclusion criteria were as follows: (1) those with symptoms of CCS and a diagnosis of CSS; (2) cervical hyperextension injury with underlying cervical spondylosis and stenosis; (3) undergoing ACAF; (4) with complete clinical data that facilitates to further comparison; (5) with complete clinical score; (6) all patients were followed up for at least 12 months after surgery.

The exclusion criteria were (1) with a history of cervical infection and tumor; (2) without a complete data of follow‐up; (3) with clinical symptoms resulting from thoracic or lumbar degenerative disease; (4) with congenital spinal deformity; (5) combined with neurological disease such as Parkinson, Alzheimer dementia, etc.; (6) combined with diabetes or other metabolic disease without regular treatment and well control; (7) with a history of psychosis, or alcoholism or drug addiction.

The sample size in the present study was determined by the number of eligible cases following the introducing of ACAF. All included patients with CCS were treated by ACAF. This study was approved by the institutional review board of the corresponding author's Hospital (SL2021021‐6), and all eligible patients signed the informed consent.

### 2.2 Clinical Evaluation

- American Spinal Injury Association (ASIA) grading system: Neurological function was assessed using the ASIA grading system (Table S1). The baseline ASIA assessment was performed within 24 h on all subjects, mainly including neurologic level of injury (NLI), ASIA motor score (AMS), ASIA sensory score (ASS) and the overall ASIA Impairment Scale (AIS) grade. A score implies complete injury and B–D scores indicate incomplete injury.
- Japanese Orthopaedic Association (JOA) score: JOA score was also used to evaluate the recovery of neurological function. This scale consists of six domain scores (motor dysfunction in the upper extremities, motor dysfunction in the lower extremities, sensory function in the upper extremities, sensory function in the trunk, sensory function in the lower extremities, and bladder function), scaled from 0 to 4, 4, 2, 2, 2, and 3, respectively, with the minimum total score being 0 and the maximum total score being 17. Higher score indicates better neurological function. The recovery rate (RR) of JOA was calculated as IR = (final JOA score − preoperative JOA score/17 − preoperative JOA score) × 100% [4].

### 2.3 Radiological Evaluation

All patients were performed x‐ray, computed tomography (CT), and magnetic resonance imaging (MRI) of the cervical spine before surgery, 3 days after surgery, and at the final follow‐up. The imaging parameters included associated pathologies, prevertebral HI, ISI, and TPR. All radiological data were independently assessed by two experienced observers, who were blinded to the group situation and patient information. Each observer measured all of the radiological parameters three times and recorded the mean values. Interclass correlation coefficients were calculated to assess the inter‐observer reliability.

**FIGURE 1:**
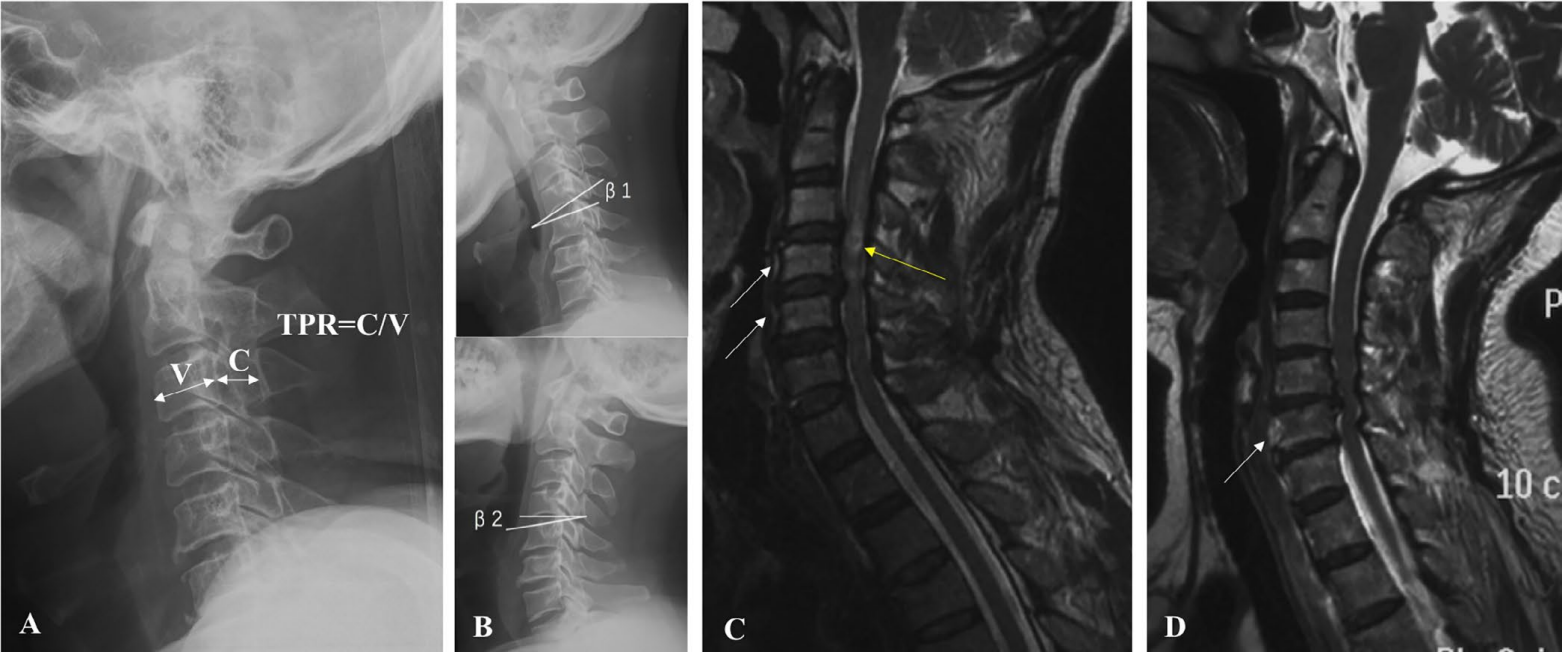
Illustration of the imaging evaluation in this study. (A) The measurement of the Torg–Pavlov ratio (TPR) by lateral x‐rays. (B) β1 is the angle on flexion view, and β2 is the angle on extension vie. Cervical instability was considered present if the angular displacement is more than 11°. (C) The ISI was shown by yellow arrow. (D) The prevertebral HI was indicated by white arrow. The level of ISI was similar to the level of prevertebral HI. HI, hyperintensity; ISI, intramedullary signal intensity.

- Plain radiographs: plain radiographs are important to delineate fractures and dislocations and provide a preliminary guide to the degree and extent of spondylosis changes.
- TPR: TPR is known as the canal‐body ratio and was measured by comparing the anteroposterior diameter of the mid‐vertebral body to the spinal canal diameter on lateral x‐rays from C3 to C6 level. A TPR of less than 0.82 has been suggested to be a marker of cervical spine canal stenosis (Figure 1A) [12].
- Cervical stability: the cervical stability was evaluated by angular displacement which is the summation of the angle β1 and angle β2 (β1 is the angle on flexion view, and β2 is the angle on extension view), and cervical instability was considered present if the angular displacement is more than 11° (Figure 1B) [13].
- CT scans: CT scans can be obtained to gain a better understanding of bony injury, to assess the degree of bony canal narrowing, and to detect injuries that are not obvious on plain radiographs.
- MRI: MRI can be useful in further assessing the presence of ISI (Figure 1C, yellow arrow), prevertebral HI (Figure 1C,D, white arrow), and the spinal cord compression. ISI and prevertebral HI frequently indicate more severe CSS.

### 2.4 Surgical Technique

**FIGURE 2:**
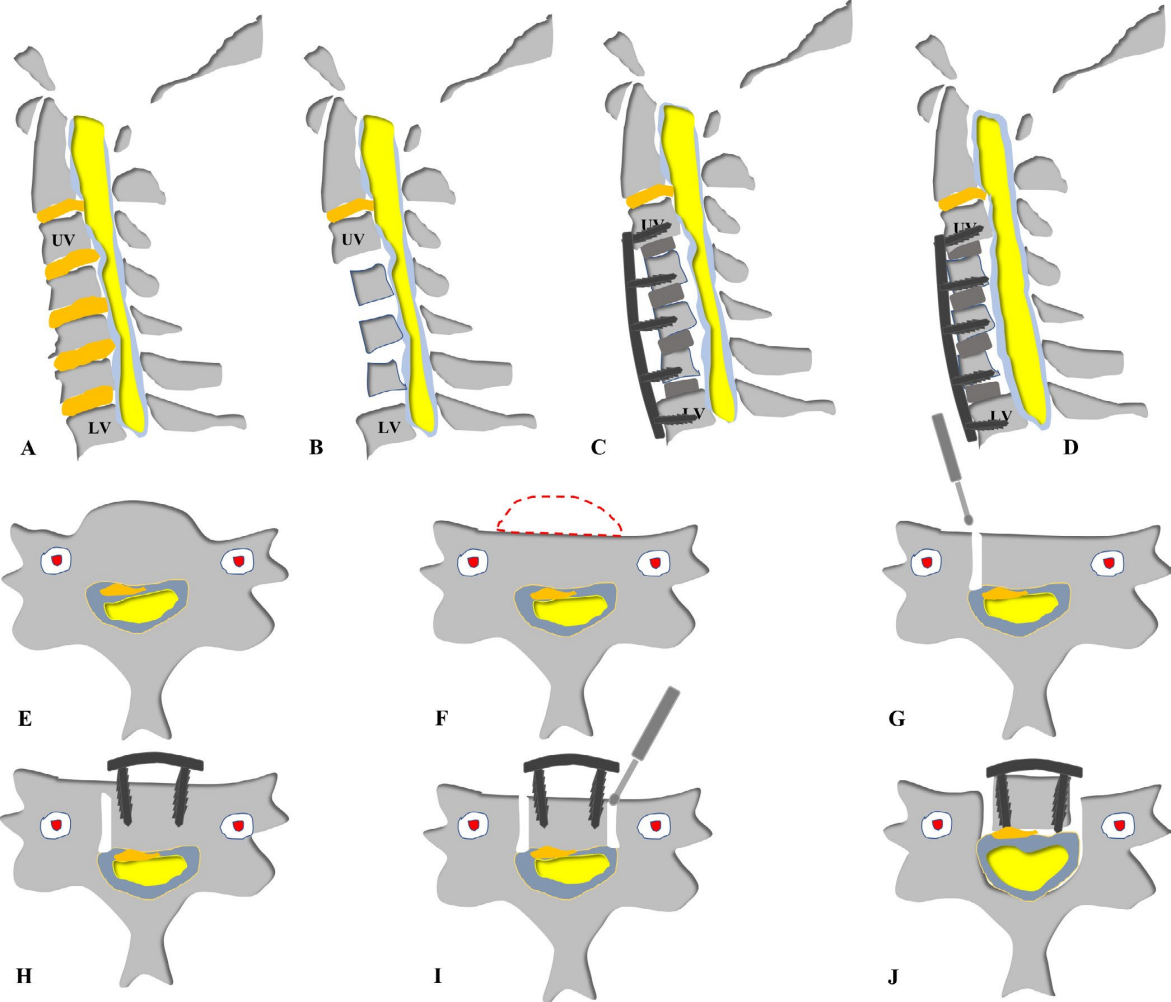
Illustration of ACAF in a sagittal (A–D) and axial (E–J) view. (A) Removal of and osteophytes of the affected segments. (B) Removal of the anterior portion of the involving vertebral bodies and the herniated disc tissues. (C) Prefixation of the cervical plate and screws, and slotting on both sides of the affected vertebral bodies. (D) Controllable antedisplacement of the vertebral body. (E) OPLL or osteophytes at the operative level. (F) Removal of the anterior portion of the vertebral body. (G) Slotting at one side lateral to the margin of OPLL or osteophytes. (H) Prefixation of cervical plate and screws. (I) Slotting at the other side lateral to the margin of OPLL or osteophytes. (J) Controllable antedisplacement of the vertebral bodies. ACAF, anterior controllable antedisplacement and fusion; OPLL, ossification of posterior longitudinal ligament.

**FIGURE 3:**
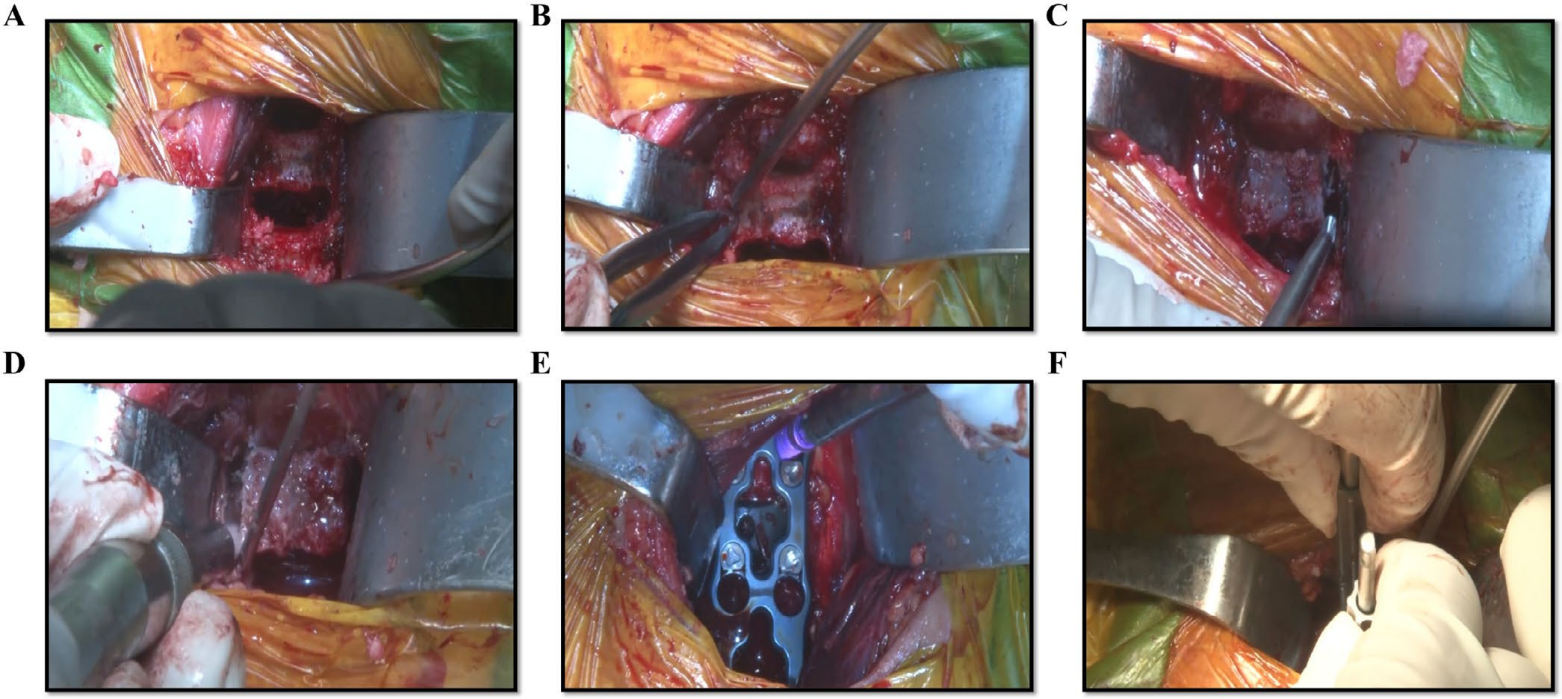
Representative images of ACAF during operation. (A) Discectomy of the involved levels. (B) Resection of the anterior vertebral bodies of the involved vertebral bodies. (C and D) Bilateral osteotomies of the involved vertebral bodies. (E) Installation of the cage and cervical plate. (F) Ante‐displacement of the involved vertebral bodies with associated pathologies.

The procedure of ACAF has been well descripted in our previous studies and is illustrated in Figure 2A–J [8, 9, 10, 11]. The surgery processes were as follows:

1. Anesthesia and position: After general endotracheal anesthesia, the patient was placed in a supine position appropriately with the neck slightly extended. The cervical spine was exposed through a standard right anterior approach, and it is helpful to note or mark the midline before elevating the longus to maintain symmetric dissection.
2. Determination of surgical level and multilevel discectomies: The surgical level was confirmed via intraoperative radiography. Routine discectomies were performed in the involved levels, and the involved levels are defined as the discs with associated pathologies and one disc superior and one disc inferior to the associated pathologies (Figure 3A).
3. Resection of posterior longitudinal ligament: After discectomies, the resection of posterior longitudinal ligament at caudal and cranial ends of involved levels was performed to facilitate the further hoisting of the affected segments (Figure 2A). In cases with the OPLL exceeding the caudal or cranial border of the associated pathologies, transection of OPLL will be required.
4. Resection of anterior vertebral bodies: Further, the proper amount of anterior vertebral bodies of the affected segments were resected by a Leksell rongeurs or a high‐speed burr according to the anteroposterior diameter of the spinal canal and the thickness of the ossified mass (Figures 2B and 3B). During the procedure, a pre‐bent anterior cervical plate can be temporarily placed to evaluate if the space between the plate and remaining vertebral body is enough for the hoisting of the OPLL.
5. Creation of bilateral longitudinal osteotomies: Following this, bilateral longitudinal osteotomies (about 3 mm of width) for isolation of the vertebrae from the surrounding bony structures were conducted using high‐speed drill, approximately at the anterior base of uncinate process (Figure 3C,D). This procedure was repeated at each affected level. Simultaneously, the intervertebral cages with autogenic bone were placed at corresponding levels.
6. Installation of cervical plate: The pre‐curved cervical plate was installed and the screws at the affected vertebrae were inserted halfway for temporary fixation (Figures 2C and 3E).
7. Antedisplacement of vertebral body‐ossified mass complex (VOC): After complete isolation of the vertebrae, the screws were gradually tightened in each vertebral body at the same pace to achieve anteriorly hoisting of the affected segments as a whole (Figures 2D and 3F).

From perspective of axial view, the OPLL or osteophytes compressed the spinal cord can be confirmed by preoperative imaging (Figure 2E). Based on thickness of OPLL or osteophytes, the anterior portion of the vertebral body was resected properly (Figure 2F). Slotting at one side lateral to the margin of OPLL or osteophytes was performed firstly (Figure 2G). Prefixation of cervical plate and insertion of screws were subsequently conducted (Figure 2H). Next, slotting at the other side lateral to the margin of OPLL or osteophytes was made (Figure 2I). A controllable antedisplacement of the vertebral bodies were final performed (Figure 2J). Subsequently, allogenic iliac bone was implanted into the bilateral longitudinal osteotomies to ensure fusion, and the bilateral longus colli were reconstructed to the anterior plate by 3–0 silk sutures to cover the graft bones. Finally, the skin was sutured layer by layer.

All patients had neurophysiologic monitoring (somatosensory‐evoked potentials), spontaneous electromyogram, and/or motor‐evoked potential intraoperatively. All patients were operated by spine surgeons who had at least 10‐year experience of spine surgery from the same surgical team. After surgery, all patients wore a Philadelphia collar for at least 3 months.

### 2.5 Statistical Analysis

Statistical analyses were performed using SPSS (Version 20.0; IBM Corp., Armonk, New York, USA). The data were presented as the mean ± standard deviation. The Mann–Whitney *U* test was used to compare the clinical outcomes preoperatively and postoperatively. Values less than 0.05 (*p* < 0.05) were considered statistically significant.

## 3 Results

### 3.1 Perioperative Characteristics

Table 1 showed the perioperative characteristics of patients in this study. Of the 21 patients, three patients were lost to follow‐up and two were excluded due to the follow‐up less than 12 months. Finally, 13 patients (7 male and 6 female) with the minimum of 12‐month follow‐up were finally enrolled in this study. The average age was 56.6 ± 12.5 years old (range, 39–74 years). Eight patients suffered CCS due to fall on their face, three due to vehicle accident, and two due to diving injuries. Surgery was undertaken as soon as possible, with some delay secondary to patient's medical conditions due to the need for preoperative clearance, or other traumatic injuries. The average delay from injury to surgery was 2.23 days (range, 1–4 days). There were 11 patients with surgical segments being four levels, and 2 patients with five surgical segments. The mean duration of follow‐up was 16.1 ± 3.5 months (range, 12–22 months). The mean operation time was 189.4 ± 26.0 min (range, 139–229 min), with the mean blood loss of 176.8 ± 31.4 mL (range, 128–234 mL).

**TABLE 1:** Demographic data of the patients.

Cases	Age/Gender	Cause of injury	Associated pathologies	ISI	Prevertebral HI	Operative day	Surgical segments	DFU (months)	Blood loss (mL)	OT (minutes)
1	66/M	Fall	HNP–OC	None	None	1	C3, C4, C5, C6	12	209	217
2	51/M	Fall	HNP	C4/5, C5/6	C5, C6	1	C4, C5, C6, C7	16	178	188
3	44/M	Diving injuries	HNP	C4, C5, C6	C4, C5	2	C3, C4, C5, C6	14	138	170
4	41/M	Diving injuries	HNP	C6/7	None	1	C3, C4, C5, C6, C7	19	163	186
5	39/M	Fall	HNP–OC	C5, C6, C7	C4/5	3	C4, C5, C6, C7, T1	13	181	190
6	73/F	Vehicle accident	HNP–OC OPLL	C5/6	C5	3	C4, C5, C6, C7	12	234	229
7	74/F	Fall	OPLL	C5	C6, C7	4	C4, C5, C6, C7	21	206	158
8	45/M	Vehicle accident	HNP	None	None	3	C4, C5, C6, C7	22	128	195
9	62/F	Fall	HNP–OC	C4/5, C5	C5	1	C3, C4, C5, C6	17	170	218
10	49/F	Vehicle accident	HNP–OC	C4, C4/5	C4, C5	2	C3, C4, C5, C6	12	139	167
11	57/F	Fall	HNP–OC	C4/5	None	3	C4, C5, C6, C7	19	159	211
12	64/M	Fall	HNP	C5/6	None	2	C3, C4, C5, C6	15	197	139
13	71/F	Fall	OPLL	C4/5, C5/6	C5	3	C4, C5, C6, C7	17	196	194

Abbreviations: DFU, duration of follow‐up; F, female; HI, hyperintensity; HNP, herniated nucleus pulposus; ISI, intramedullary signal intensity; M, male; OC, osteophyte complexes; OPLL, ossification of posterior longitudinal ligament; OT, operation time.

### 3.2 Radiological Outcomes

**FIGURE 4:**
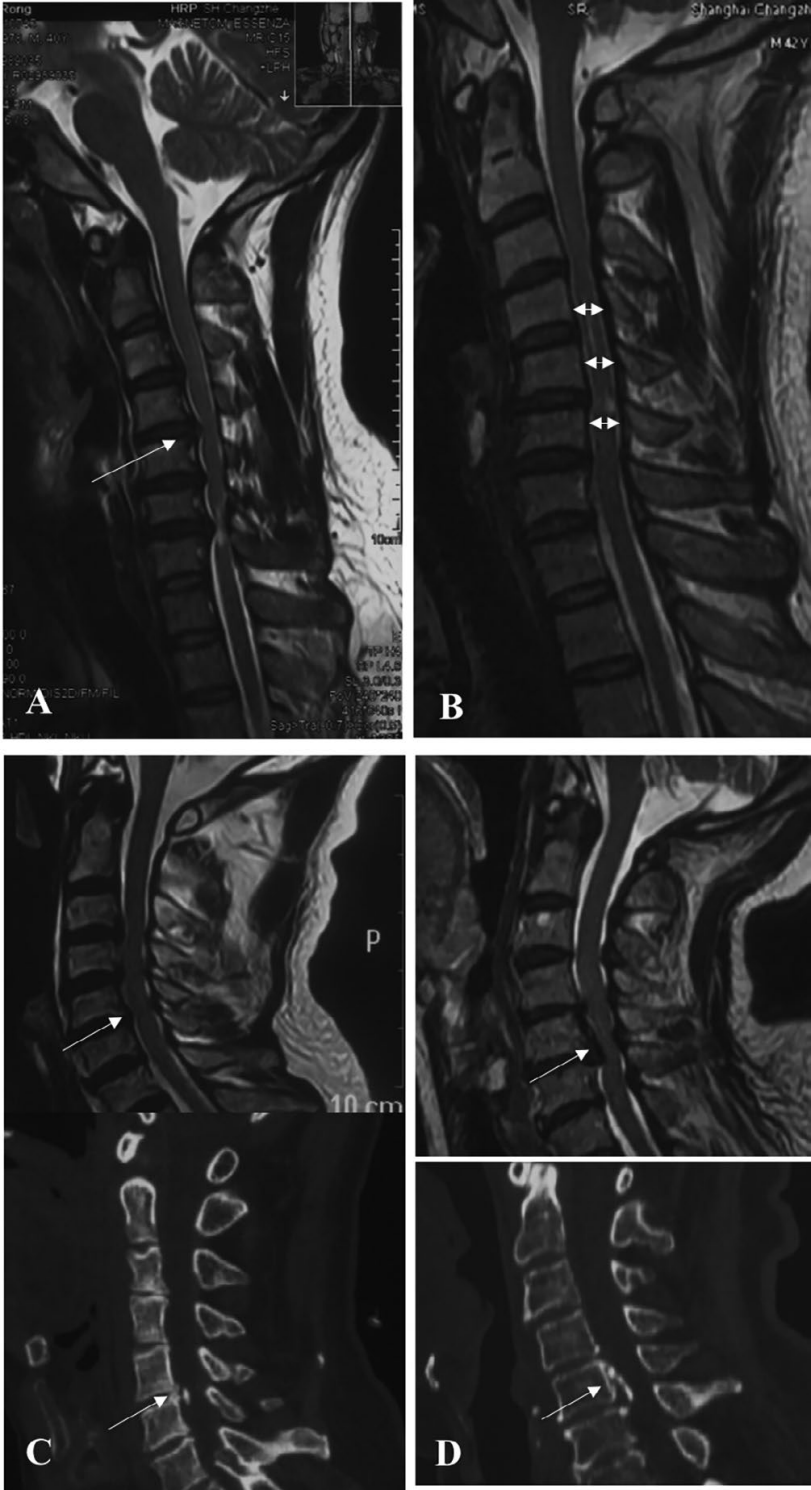
Representative images of HNP (A), CS (B), HNP‐osteophyte complexes, and OPLL (local type [C and D]). CS, cervical stenosis; HNP, herniated nucleus pulposus; OPLL, ossification of the posterior longitudinal ligament.

The inter‐observer correlation coefficients were 0.86, which showed strong agreement among the measurements. 76.9% (10 of 13) patients were observed to have cervical stenosis indicated by TPR (Table 2). All patients acquired significant improvement of TPR at decompression segments after surgery compared to preoperation (Table 2, bold). In terms of prevertebral HI and ISI, eight patients had prevertebral HI and segments from C4 to C6 were the most affected region. Associated pathologies were HNP in five patients, OPLL in three cases, and HNP‐osteophyte complexes (HNP‐OC) in six patients (Figure 4). Eleven patients had ISI before surgery, with the most common segment at C5 level. All patients acquired good bony fusion at the final follow‐up revealed by CT.

**TABLE 2:** Changes of Torg–Pavlov ratio from C3 to C6 before and after surgery.

Cases	C3		C4		C5		C6		Decompression segments
	Pre.	Post.	Pre.	Post.	Pre.	Post.	Pre.	Post.	
1	0.676	0.676	**0.640**	**1.088**	**0.618**	**1.001**	0.618	0.618	C4, C5
2	0.950	0.950	0.864	0.864	**0.834**	**1.289**	**0.910**	**1.280**	C5, C6
3	0.500	0.500	**0.492**	**0.556**	**0.649**	**1.090**	0.592	0.592	C4, C5
4	0.680	0.680	**0.801**	**0.798**	**0.732**	**0.850**	**0.712**	**0.833**	C4, C5, C6
5	0.667	0.667	0.750	0.750	**0.649**	**1.541**	**0.592**	**1.605**	C5, C6, C7
6	1.000	1.000	1.000	1.000	**1.029**	**1.782**	**1.065**	**1.272**	C5, C6
7	0.859	0.859	0.909	0.909	**0.711**	**1.041**	**0.767**	**0.974**	C5, C6
8	0.890	0.890	0.887	0.887	**0.643**	**1.288**	**0.701**	**1.570**	C5, C6
9	0.850	0.850	**0.601**	**1.120**	**0.699**	**1.391**	0.901	0.901	C4, C5
10	0.633	0.633	**0.598**	**1.354**	**0.679**	**1.098**	0.802	0.802	C4, C5
11	1.100	1.100	0.975	0.975	**0.729**	**1.782**	**0.679**	**1.284**	C5, C6
12	0.700	0.700	**0.689**	**1.320**	**0.665**	**1.295**	0.817	0.817	C4, C5
13	0.959	0.959	1.032	1.032	**1.121**	**1.783**	**0.983**	**1.292**	C5, C6

### 3.3 Clinical Outcomes

The neurological outcomes were summarized in Table 3. Before surgery, there were two patients with the ASIA grade of B, six with C, and five with D. However, at the final follow‐up, 13 patients were improved to E. In terms of JOA score of the patients, the mean JOA score of patients was 10.1 ± 3.2 before surgery, while at the final follow‐up the mean JOA score was 15.4 ± 1.0. The final averaged recovery rate was 77.0% ± 12.0%. In terms of complications, two patients experienced postoperative dysphagia, two patients had hoarseness, and one patient suffered postoperative hematoma. In fact, all these complications are the common complications in cervical anterior approach, such as ACDF and ACCF. No serious surgery‐related complications, such as CSF leak and transient C5 root injury, were observed during the follow‐up in these 16 patients, which we attributed to the whole operation manipulation of ACAF being conducted outside the spinal canal.

**TABLE 3:** Changes of neurological function of the patients in this study.

Cases	ASIA grade			JOA score			Final recovery rate
	Pre.	Post.	Final	Pre.	Post.	Final	
1	D	D	E	9	11	14	0.63
2	B	D	E	9	14	16	0.88
3	C	E	E	14	16	16	0.67
4	D	E	E	13	16	16	0.75
5	C	E	E	16	17	17	1.00
6	C	E	E	13	14	16	0.75
7	C	D	E	5	13	16	0.92
8	C	D	E	7	12	14	0.70
9	C	E	E	11	15	15	0.67
10	D	E	E	10	16	16	0.86
11	D	E	E	8	14	15	0.78
12	B	D	E	7	13	15	0.80
13	D	D	E	9	14	14	0.63

Abbreviations: ASIA, American Spinal Injury Association; JOA, Japanese Orthopaedic Association.

### 3.4 Representative Cases

**FIGURE 5:**
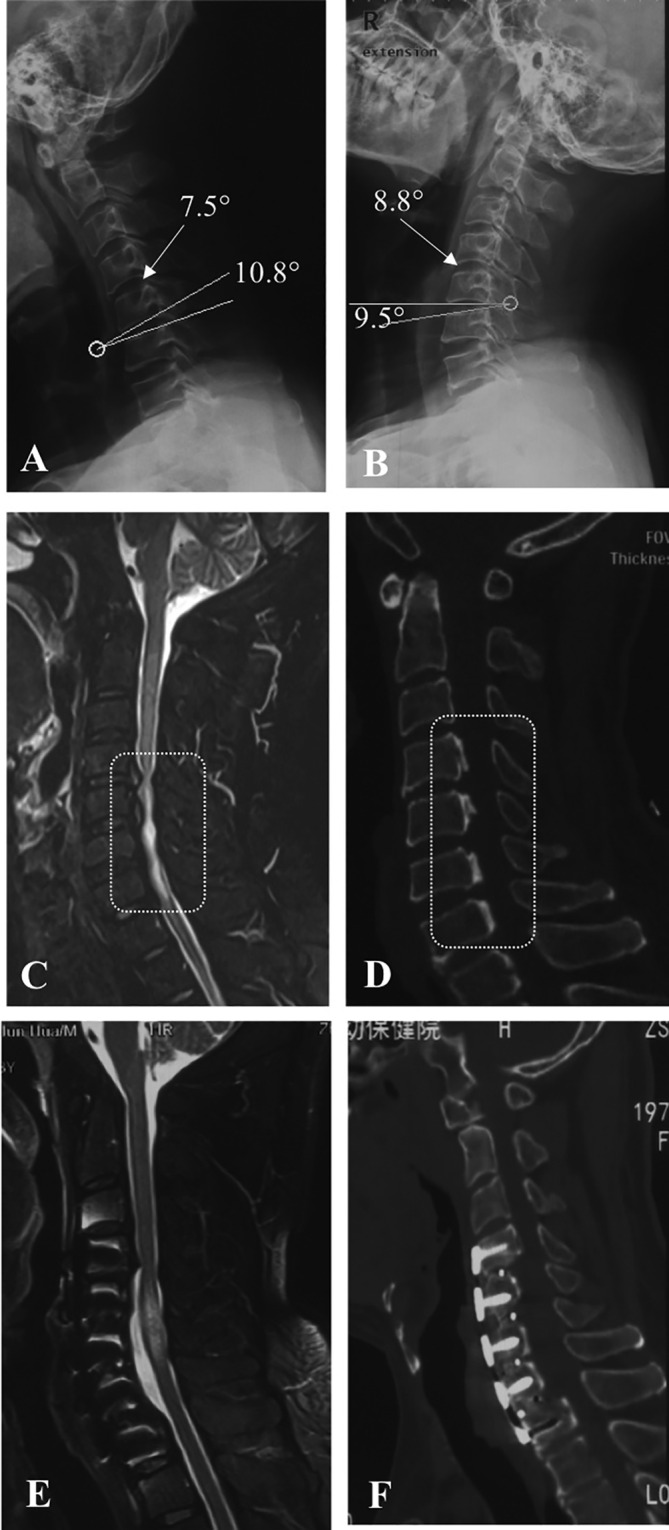
Case 3: (A) Preoperative radiograph at over flexion position. (B) Preoperative radiograph at over extension position. Dynamic cervical lateral radiograph revealing instability at C4/5 (16.3° > 11°) and C5/6 (20.4° > 11°) with developmental narrow canal. (C) Sagittal T2‐weighted MRI showing multilevel cord compression (dotted square) and prevertebral hyperintensity (C4/5). (D) Sagittal CT showing segmental OPLL from C4 to C7 (dotted square). (E) Sagittal MRI after surgery demonstrating good decompression and expansion of spinal cord and decreased intensity signal of ISI on C5–C6. (F) Postoperative CT indicating satisfactory enlargement of spinal canal at C5–C7 levels (Table 2). OPLL, ossification of the posterior longitudinal ligament.

Case 3 (long surgical segments): A 39‐year‐old male patient with hyperextension injury was admitted to our institution due to fall from the stairs. Preoperative imaging showed that he had cervical instability and cervical canal stenosis at C5 (0.649) and C6 (0.592) levels (Figure 5A,B). he had previous herniated nucleus pulposus (HNP) associated osteophyte from C4 to C7 with ISI and prevertebral HI at C4/5 level (Figure 5C,D). The patents underwent ACAF from C4 to T1 3 days after admission. Posterior MRI suggested that his spinal cord was decompressed sufficiently and his stenotic cervical canal was also enlarged (Figure 5E,F). At the final follow‐up, his ASIA score improved to E and his JOA score recovered to 17.

## 4 Discussion

### 4.1 The Principal Results of This Study

In this present study, a total of 13 patients with CCS were enrolled and all patients accepted the treatment of ACAF. At the final follow‐up of at least 12 months, all patients acquired satisfactory recovery of neurological function. The results of this study preliminarily suggested ACAF can become a reliable alternative for treating CCS patients due to hyperextension injury with underlying cervical spondylosis and stenosis.

### 4.2 Underlying Cervical Spondylosis Correlated With Severe Damage and Bad Recovery in Patients With CCS

It has been reported that more than 90% of patients with CCS aged over 40 years suffer underlying cervical spine diseases, such as spondylosis with osteophyte formation, canal stenosis, and ossification of the posterior longitudinal ligament (OPLL) [14]. This present study demonstrated similar results that there were 13 (81.3%) patients with developmental canal stenosis, and all patients aged more than 40 years. Therefore, patients with underlying cervical spine diseases frequently suffer from more severe neural damages and worse recovery.

Anatomically, the anterior longitudinal ligament bears the majority of tensile force, discs support the major axial loading, and posterior ligaments combined with other structures control the flexion motion of the cervical spine. When individual suffering horizontal violence such as car collisions or falls, the load applied to cervical segments will be beyond normal physiological range, which can induce a suddenly simultaneous squeezing or pinching to the spinal cord both anteriorly and posteriorly [15]. However, under the circumstance, individuals with underlying cervical spinal stenosis or other stenosis‐related diseases will be susceptible to CCS owing to the limited reserved space in spinal canal [16, 17]. As indicated in this study, the most compressed level was the most frequent injury level due to the limited buffer space. In addition, an investigation suggested that a bimodal distribution of age in CCS patients, with the 50 years representing the median age [18, 19]. In our study, the causes of injury varied between age societies, with simple falls from a standing position being the most frequent cause in those aged over 50 years (68.8%), whereas high‐energy trauma (vehicle accident or diving injuries) being more common in those more 50 years (31.2%). Other than injuries of neural elements, the ligaments and intervertebral discs injuries and cervical instability are also important issues which should be considered. What is more, the damage of the cord is not always confined at the level of intervertebral disc [20]. Therefore, a timely and reasonable clinical intervention is critical for preserve and restore patients' neurological function for those with underlying cervical spondylosis.

### 4.3 Traditional Management of CCS

Schneider et al. initially suggested such patients would be able to recover spontaneously and recommended conservative treatment [21]. In fact, most patients had good recovery with proper medical management, such as intravenously administered methylprednisolone and blood pressure augmentation in intensive care [5, 22]. Early mobilization and rehabilitation with physical therapy and occupational therapy are essential once the patient is medically stable. However, there are still many patients, especially those with underlying cervical spondylosis or stenosis, who experience plateau and even deteriorating neurological function during conservative treatment [1]. Therefore, surgical decompression will be recommended for such patients [3]. A multi‐center study enrolling 313 patients with acute cervical SCI by Fehlings et al. has found that early decompression surgery prior to 24 h was associated with better neurological recovery and less complications as compared to delayed surgery procedure [23]. As a result, prompt and effective surgical decompression are significantly required to release the spinal cord and to prevent further cervical degenerative diseases, which has also been encouraged in the clinical practice of our institution.

Anterior procedures could directly deal with protruding intervertebral disc or ossified mass to release anterior compression of the spinal cord. ACDF is commonly applied to single‐ or two‐level compressed segments, with good cervical stability [24]. However, the decompression effect will be limited when the lesion is behind vertebral bodies. In addition, surgery‐related complications such as pseudarthrosis and deep wound infection will be high in cases with multilevel segments [25, 26, 27]. ACCF has been frequently used for patients with lesion behind vertebral bodies, especially for patients with compression mainly existed at ventral cord and cervical instability. Nevertheless, this technique is highly technically demanding, and perioperative complications, such as SCI and CSF leakage, is relatively high in patients with multilevel cervical spondylosis or stenosis [28]. Additionally, for patients with CCS due to severe multilevel cervical spondylosis or stenosis, ACCF may be limited and long titanium mesh would increase the risk of nonunion, subsidence, and graft failure [29]. Therefore, traditional anterior approach is suitable for patients with potential cervical stenosis below three level, whereas for longer segments, surgery‐related complications will affect the clinical effects.

Posterior approach can decompress the spinal cord directly by posteriorly floating the cord, and in recent years, it has been also employed in cervical trauma. However, posterior approach cannot correct the significant kyphotic deformity or deal with anteriorly located lesions [30]. The cervical spine distributes 36% of compressive loads through the vertebral bodies, whereas 64% is transmitted to the posterior elements and the resection of laminae was shown to alter axial load applied on anterior column of cervical vertebral body [31]. Nowinski and colleagues have reported that the stability of cervical spine would be adversely affected by the extend of facetectomy and the number of laminae resected [32]. Therefore, patients with laminectomy without fusion may have higher risk in developing cervical instability and adjacent degeneration diseases. In addition, excessive damaging paravertebral muscles may contribute to cervical instability and even kyphosis deformity [33]. Laminoplasty, by leaving the posterior structures in situ, is believed to mitigate the development of kyphosis without being “rigid” and causing degeneration of adjacent levels [34]. However, for patients with CCS, it is necessary to restore the cervical stability and posterior laminoplasty cannot achieve this point. In addition, persistent cervical instability will also increase the risk of stenosis progression.

The combined anteroposterior approach, such as laminoplasty or laminectomy combined with ACCF and ACDF, is also suggested for the treatment of CCS patients with cervical stenosis. One‐or two‐ stage procedure could significantly extend the spinal canal area ventrally and dorsally as well as restore cervical alignment. However, this procedure is highly technically demanding and could lead to longer surgical duration, more surgical damage (such as more blood loss or nerve injury) and more postoperative complications, such as pseudarthrosis, which is not beneficial for the prognosis of CCS patients with injured spinal cord [35, 36]. In our institution, this type of technique is not routinely recommended.

### 4.4 ACAF in the Treatment of CCS With Underlying Cervical Degeneration‐Related Diseases

An optimal surgical procedure for CCS patients with cervical spondylosis or stenosis should reconstruct the volume of the bony spinal canal, maintain its sagittal alignment, and result in fewer complications in only one approach [8, 9, 10, 11]. ACAF, which has been initially proposed to treat patients with cervical OPLL, can achieve all these goals. This technique can enlarge the spinal canal by anteriorly ante‐displacing the affected segments and associated pathologies as a whole, which can create the least disturbance to the dura mater and spinal cord. Additionally, the hoisted vertebrae‐OPLL complex (VOC) can serve as a part of the anterior column of cervical spine, which are instrumental in the reconstruction of cervical stability and the neurological recovery. In this study, almost all patients were found to have cervical instability via preoperative imaging. However, all patients treated by ACAF in this study acquired satisfactory restoration of cervical stability. In terms of stability, the superiority of ACAF over ACCF has proved in patients with cervical myelopathy caused by spondylosis or OPLL. In previous studies, ACAF demonstrated faster bone fusion than ACDF and ACCF [37, 38]. More contact surfaces and sufficient bone marrow exposure by conducting bilateral osteotomies might be the major factors for faster bone fusion after ACAF. More importantly, ACAF lead to lesser subsidence than ACCF at the 1‐year follow‐up. Its incidence of implant‐related complication was lower than that of ACCF, including graft migration, displacement, and pseudarthrosis [37, 38, 39]. According to an in vitro biomechanical study, we found that the biomechanical stability levels for ACAF and ACDF were similar and were both significantly greater than that of ACCF [39]. For ACCF, a long lever arm caused by long strut titanium mesh and plate fixations would increase the stress of caudal screws, thus making vulnerable to graft migration. By contrast, multiple fixation points of ACAF could shorten the lever arm, and thus stabilizing the construct, which would result in less subsidence than that in ACCF. As shown in Table 2, all patients acquired significant improvement of TPR at their decompression segments after surgery, which indicated that ACAF can be a good option for patients with cervical stenosis. Based on the results of this present study, the surgical segments covered C3, C4, C5, C6, or C7, with the most common segments be C5 level. In addition, the associated pathologies in patients included HNP, HNP‐osteophyte complex, cervical spinal canal stenosis, and OPLL (Figure 3). All patients acquired satisfactory reconstruction of spinal canal and neurological function at the final follow‐up. Therefore, we preliminarily proposed the surgical indications of ACAF for patients with CCS as follows: (1) patients with associated pathologies, including cervical spinal canal stenosis, HNP, HNP‐osteophyte complex, and OPLL; (2) patients with or without ISI; (3) patients with or without prevertebral HI; (4) surgical segments including C3, C4, C5, C6, or C7; (5) patients with underlying lesion involved three or more levels. As for the contraindications, we believed that all the contraindications for hyperextension injury were consistent with those for OPLL, as presented in exclusion criteria. However, due to the limited sample size, we cannot conclude which segment or which associated pathology will benefit most from ACAF. Studies with more cases and longer duration of follow‐up will be required. This study was a preliminary investigation to explore the surgical effect of ACAF on patients with hyperextension injury.

Compared with traditional anterior or posterior approach, the spinal cord remains in its original position and can be kept in a good lordosis after ACAF, which we named “in situ decompression” [40]. The concept of “in situ decompression” ensures no iatrogenic tension imposed on the spinal cord resulted from anterior or posterior shift of the cord. A study by Lau et al. suggested greater cervical lordosis was associated with better clinical outcomes, especially for lordosis greater than 20° [6]. Although no study has claimed that the spinal cord must be kept in normal position, good curvature, and even physical lordosis, and yet in theory it should be. In this present study, all patients were performed by ACAF acquired good restoration of cervical spine curvature and spinal cord alignment. What is more, at the final follow‐up, all patients had improved neurological function, including 13 patients were improved to E. The mean JOA score of patients was improved from 10.1 ± 3.2 before surgery to 15.4 ± 1.0 at the final follow‐up, with the final recovery rate of 77.0% ± 12.0%. However, longer duration of follow‐up is still required to validate the long‐term clinical outcomes of ACAF.

The technique of ACAF involves removing part of the vertebral body to create space for ventral antedisplacement of the ossified ligament. Compared to traditional anterior surgery, the holding power of screws on the treated vertebral segment is relatively weaker. If the patient has severe osteoporosis, the incidence of instability in the screws of the distracted vertebral segment may be higher than that in traditional surgeries. In our clinical practice, we will evaluate the osteoporosis state of all the patients before operation. Based on our clinical experience, for patients with slight and moderate osteoporosis, ACAF could achieve successful antedisplacement of the ossified ligament. However, for patients with severe osteoporosis, during screw placement, we will inject bone cement into the screw tract to enhance the holding power of the screws on the treated vertebral segment. Therefore, osteoporosis can be considered as a relative contraindication for ACAF. In addition, the learning curve of ACAF should be described here for better knowledge of this technique. In fact, we have published a study to quantify a surgeon's learning curve for ACAF and the effect of surgeon experience on postoperative outcomes [41]. In this study, we found that about 29 cases were needed to achieve mastery of ACAF. Once mastered, the surgeon could deal with various OPLL presentations in a universal way regardless of condition complexity and number of surgical levels. Bilateral osteotomies were the most difficult part of ACAF and produced the greatest reduction in time after mastery. We found a close association between specific errors of surgical procedure for ACAF and surgeon experience. Furthermore, certain complications caused by these errors should be on the alert during the early phase of learning ACAF, including CSF leakage, C5 palsy and incomplete decompression. We wish the results will provide useful information for spinal surgeons to learn this form of surgery.

In terms of the ossified ligament, despite the absence of long‐term follow‐up studies that directly compare the progression of ossifications following ACAF versus posterior surgery, Nevertheless, numerous studies have documented the occurrence of thickening of the ossification and the emergence of new spinal cord compression symptoms subsequent to posterior surgery [42, 43]. However, a surprising finding in the study reports on ACAF is the tendency of the ossified group to remodel into the anterior wall of the spinal canal after surgery [44]. This is evidenced by the transformation of ectopic bone into part of the vertebral bone, with the ossified mass not growing toward the spinal cord side during our follow‐up, and the relevant follow‐up results are currently being compiled. It has been reported that the progression of ossification after posterior surgery occurs due to decreased postoperative cervical stability and mechanical stress stimulation [43, 45, 46]. In contrast, the ACAF procedure can greatly improve the stability of the operated segments due to excellent internal fixation and effective postoperative fusion, which creates favorable conditions for the remodeling of the postoperative ossification. It can be postulated that the outcome of postoperative ossification following ACAF may diverge significantly from that of posterior surgery. It is reasonable to hypothesize that the reappearance of neurological symptoms due to ossification growth will not occur.

### 4.5 Strengths and Limitations

Our study has the following strengths: (1) despite limited sample sizes, the results of this study demonstrated that ACAF can be a new alternative in the treatment of patients with CCS, especially those with cervical stenosis; (2) the follow‐up was conducted for a minimum duration of 12 months. The satisfactory outcomes encouraged us to performed further research to ensure the therapeutic effects of ACAF on CCS; (3) the whole operation manipulation of ACAF was conducted outside the spinal canal, which could significantly minimize the risk of spinal cord or root injury. As shown in results of the study, no patients experienced serious surgery‐related complications, such as CSF leak and transient C5 root injury, during the follow‐up, which could ensure the non‐complication recovery in patients.

However, this present study has several limitations. First, due to the limitations of the disease itself and study duration, the sample size was small and the duration of follow‐up was relatively short. In fact, this retrospective study was preliminary clinical research, and thus this clinical therapy was firstly performed by a single physician who can operated ACAF skillfully. As the results shown, most of the patients acquired satisfactory outcomes after ACAF, which will encourage us to conduct a study with more physicians or even multiple centers with longer duration of follow‐up in the future to further validate the generalizability of the results. Second, we did not conduct a comparative study with patients treated by other techniques, which will further expand the application scope of ACAF and provide a new alternative for CCS patients with underlying cervical spondylosis or stenosis. Second, due to the limitations of the patient size, we cannot outline a comprehensive algorithm which types of surgeries and how they are chosen with different clinical, and radiological criteria for a CCS patient. However, based on this study, we can give a reference for the treatment of those patients. If the cervical stenosis of patient is less than three segments, ACAF can be an alternative, whereas if the involved segments are more than three levels, posterior surgery will be recommended due to the safety. In addition, for those with nucleus pulposus herniation or osteophyte, ACAF or ACDF will be suggested.

## 5 Conclusion

ACAF can be a good option to treat CCS patients with underlying cervical spondylosis and stenosis.

## Author Contributions

Data collection and writing – original draft, investigation: Shuangxi Sun, Yingying Miao, and Tao Xu. Data curation: Kaiqiang Sun and Yijuan Lu. Conceptualization, project administration, writing – review and editing: Jingchuan Sun, Jiuyi Sun, and Jiangang Shi. All authors reviewed this manuscript and approved it.

## Conflicts of Interest

The authors declare no conflicts of interest.

## Supporting information


**Table S1:** American Spinal Injury Association scale (ASIA) Impairment Scale (AIS) grade according to International Standards for Neurological Classification‐1 (ISNC‐1).

## Data Availability

The data that support the findings of this study are available from the corresponding author upon reasonable request.
