# Supplementary material for: Anterior Controllable Antedisplacement and Fusion (ACAF) for Cervical Stenosis Patients With Hyperextension Injury: A Retrospective Study
Source: Orthop Surg. 2024 Dec 12;17(2):500–12. doi: 10.1111/os.14319 (PMC11787972; doi:10.1111/os.14319)
Supplement: Supplementary file 1 — Table S1: American Spinal Injury Association scale (ASIA) Impairment Scale (AIS) grade according to International Standards for Neurological Classification‐1 (ISNC‐1). [file OS-17-500-s001.docx]

Supplementary table 1 American Spinal Injury Association scale (ASIA) Impairment Scale (AIS) grade according to International Standards for Neurological Classification-1 (ISNC-1).

| Grade | Symptoms |
| --- | --- |
| A | Complete. No sensory or motor function is preserved below the level of injury |
| B | Sensory incomplete. Sensory but no motor function is preserved below the motor level on either side of the body |
| C | Motor incomplete. Motor function is preserved, but less than half of key muscle functions below the single neurologic level of injury (NLI) have a muscle grade <3 |
| D | Motor incomplete. Motor incomplete status as defined above, with at least half (or more) of key muscle functions below the single NLI having a muscle grade ≥3 |
| E | Normal |
